# Incidence of rhabdomyolysis occurrence in psychoactive substances intoxication: a systematic review and meta-analysis

**DOI:** 10.1038/s41598-023-45031-4

**Published:** 2023-10-17

**Authors:** Alireza Amanollahi, Tannaz Babeveynezhad, Mohsen Sedighi, Shahin Shadnia, Sadaf Akbari, Mahbobeh Taheri, Mahboobeh Besharatpour, Goljamal Jorjani, Elham Salehian, Koorosh Etemad, Yadollah Mehrabi

**Affiliations:** 1 Department of Epidemiology, School of Public Health and Safety, Shahid Beheshti University of Medical Sciences, Tehran, Iran; 2 Pharmaceutical Sciences Branch, Tehran Azad University, Tehran, Iran; 3 Trauma and Injury Research Center, Iran University of Medical Sciences, Tehran, Iran; 4 Department of Clinical Toxicology, Loghman Hakim Hospital, Shahid Beheshti University of Medical Sciences, Tehran, Iran; 5 Department of Internal Medicine, Division of Nephrology, Carver College of Medicine, University of Iowa, Iowa City, IA USA; 6 Skull Base Research Center, Loghman Hakim Hospital, Shahid Beheshti University of Medical Sciences, Tehran, Iran; 7 Resources Development Deputy, Shahid Beheshti University of Medical Sciences, Tehran, Iran

**Keywords:** Kidney, Toxicology

## Abstract

Rhabdomyolysis is a potentially life-threatening condition induced by diverse mechanisms including drugs and toxins. We aimed to investigate the incidence of rhabdomyolysis occurrence in intoxicated patients with psychoactive substances. In this review, three databases (PubMed, Scopus, Web of Science) and search engine (Google Scholar) were searched by various keywords. After the screening of retrieved documents, related data of included studies were extracted and analyzed with weighted mean difference (WMD) in random effect model. The highest incidence of rhabdomyolysis was observed in intoxication with heroin (57.2 [95% CI 22.6–91.8]), amphetamines (30.5 [95% CI 22.6–38.5]), and cocaine (26.6 [95% CI 11.1–42.1]). The pooled effect size for blood urea nitrogen (WMD = 8.78, p = 0.002), creatinine (WMD = 0.44, p < 0.001), and creatinine phosphokinase (WMD = 2590.9, p < 0.001) was high in patients with rhabdomyolysis compared to patients without rhabdomyolysis. Our results showed a high incidence of rhabdomyolysis induced by psychoactive substance intoxication in ICU patients when compared to total wards. Also, the incidence of rhabdomyolysis occurrence was high in ICU patients with heroin and amphetamine intoxication. Therefore, clinicians should anticipate this complication, monitor for rhabdomyolysis, and institute appropriate treatment protocols early in the patient’s clinical course.

## Introduction

Psychoactive substances affect thinking, emotion, mood and behavior, and consciousness after being consumed and are classified into central nervous system depressants (ethanol, opioids, cannabis), central nervous system stimulants (amphetamines, cocaine), hallucinogens (LSD), and empathogens (ecstasy)^1^. Nausea, vomiting, agitation, anxiety, and drowsiness are the most common adverse effects of psychoactive drugs but serious conditions such as psychosis, delirium, seizure, cardiotoxicity, severe lung injury, and acute kidney injury (AKI) due to rhabdomyolysis have seen reported in abusers^2,3^.

Previous studies have documented drug induced-rhabdomyolysis owing to the overuse of methanol, ethanol, methadone, opioid, cocaine, amphetamine, methamphetamine, ecstasy, synthetic cannabinoids, heroin, and tramadol^4,5^. Rhabdomyolysis is a syndrome characterized by muscle necrosis and the release of intracellular muscle constituents into circulation^6^. Creatine Phosphokinase (CPK) level commonly increases and muscle pain and myoglobinuria may be identified. The severity of rhabdomyolysis ranges from asymptomatic elevation in serum muscle enzymes to life-threatening conditions associated with extreme enzyme elevation, electrolyte imbalance, and acute kidney injury (AKI)^7^.

Rhabdomyolysis has been reported in a growing number of studies as one of the worst results of drug poisoning but with different incidences^8^. For instance, the incidence of cocaine-induced rhabdomyolysis is reported from 25^9^ to 46.7%^10^ and this value for methadone is between 14.7^11^ and 34.6%^12^.

Considering the increasing use of psychoactive substances in recent years and little known about drug-induced rhabdomyolysis in abusers, screening and assessing the incidence of rhabdomyolysis and proper management is essential. For this reason, we systematically reviewed the international databases in this study and the results of related papers were pooled regarding the incidence of rhabdomyolysis in hospitalized patients with intoxication.

## Methods

### Study design

This systematic review and meta-analysis study was according to the Preferred Reporting Items in Systematic Reviews and Meta-Analyses (PRISMA) guideline^13^, and protocol of the study was registered in PROSPERO (CRD42022326206).

### Search strategy

To find related publications, a combination of related keywords was used in databases and search engine including PubMed, Scopus, Web of Science, and Google Scholar. In addition, we used a manual search to develop literature search, references of selected studies, and citations of studies. The final search was updated on July 15, 2022 before data analysis. The keywords used included a combination of suggested words by Medical Subject Heading (MeSH) and other related words, as represented in details in Table 1. Finally, three limitations in research including human studies, publication date (2000–2022), and English language studies were applied. All processes related to the literature search were done independently by two researchers (AA and ES).

**Table 1:** The search strategy in all databases/search engine.

PubMed	(rhabdomyolys*[tw] OR rhabdomyolysis[mh] OR "creatine phosphokinase"[tw] OR "creatine kinase"[tw] OR cpk[tw] OR ck[tw]) AND ("toxic*"[tw] OR "toxic actions"[mh] OR overdose*[tw] OR drug overdose[mh] OR opiate overdose[mh] OR abuse[tw] OR Substance-Related Disorders[mh] OR poisons[mh] OR poisoning[tw] OR Poisoning[mh] OR intoxication[tw] OR intoxicat*[tw]) AND (opioid*[tw] OR opium*[tw] OR cannabis[tw] OR Marijuana[tw] OR Heroin[tw] OR amphetamine*[tw] OR methamphetamine*[tw] OR ecstasy[tw] OR MDMA[tw] OR methadone[tw] OR tramadol[tw] OR ethanol[tw] OR methanol[tw] OR "Alcoholic Intoxication"[mh] OR synthetic cannabinoid*[tw] OR psychoactive substance*[tw]) NOT (review[tiab] OR review[pt])
Scopus	((ALL (overdose) OR ALL (abuse) OR ALL (poison) OR ALL (poisoning) OR ALL (intoxication))) AND ((ALL (opioid) OR ALL (opium) OR ALL (cannabis) OR ALL (marijuana) OR ALL (heroin) OR ALL (amphetamine$) OR ALL (methamphetamine) OR ALL (ecstasy) OR ALL (mdma) OR ALL (methadone) OR ALL (tramadol) OR ALL (cocaine) OR ALL (methanol) OR ALL (ethanol) OR ALL (synthetic AND cannabinoid) OR ALL (psychoactive AND substance))) AND ((ALL (rhabdomyolysis) OR ALL (rhabdomyolyse) OR ALL (creatine phosphokinase) OR ALL (creatine kinase))) AND (LIMIT-TO (DOCTYPE, "le") OR LIMIT-TO (DOCTYPE, "sh") OR LIMIT-TO (DOCTYPE, "ed") OR LIMIT-TO (DOCTYPE, "ar") OR LIMIT-TO (DOCTYPE, "cp"))
Web of sciences	((TS = (opioid OR opium OR cannabis OR marijuana OR heroin OR amphetamine OR methamphetamine OR ecstasy OR MDMA OR methadone OR tramadol OR cocaine OR methanol OR ethanol OR synthetic cannabinoids OR psychoactive substance)) AND (TS = (overdose OR abuse OR poison OR poisoning OR intoxication OR toxic)) AND (TS = (rhabdomyolysis OR rhabdomyolyse OR creatine phosphokinase OR creatine kinase)))
Google Scholar	This search strategy was repeated for all psychoactives and 300 initial results were reviewed; ((poisoning OR intoxication) AND (opioid) AND (rhabdomyolysis OR creatine kinase))

### Eligibility criteria

Eligibility criteria were elaborated based on the PICO structure (Population, Intervention\ Exposure, Comparator, and Outcomes), and the studied population under meta-analysis were intoxicated patients hospitalized in the intensive care unit (ICU) or general wards due to poisoning, overdose, and abuse and were either conscious or unconscious. Intoxicated patients following enteral, parenteral, and inhalational use of methadone, cocaine, heroin, tramadol, amphetamine, methamphetamine, ecstasy, MDMA (3,4-methylenedioxyamphetamine), opioid, synthetic cannabinoids, methanol, and ethanol were included in the study. Mono-intoxication means patients who were intoxicated with a single substance and multi-intoxication means patients who had co-exposure and concomitant substances were detected in their drug screen. The research question of the study was occurrence of rhabdomyolysis among hospitalized intoxicated patients. Exclusion criteria were case reports and case series with less than 5 samples, review or editorial articles, none English language manuscripts, and studies on children.

### Study selection, data items, data collection

Retrieved observational studies from selected databases with relevant exposures were imported into EndNote citation management software. After removing duplicate studies, title and abstract of remained studies were screened and data extraction was done by two independent researchers (TB and SA). Data extraction forms contained the author's name, year, age, gender, country, continent, study design, type of psychoactive drug, sample size, type of hospitalization wards (ICU/total wards), multi-intoxication (yes/no), dose of substance, and patient’s medical history. Also, subgroup analyses for hospitalization ward, multi-intoxication, and geographical area (based on World Health Organization regions) was performed. In subgroup analysis, ICU means studies that included only ICU patients and total wards mean studies reporting total patients hospitalized in ICU and general wards. Also, poisoning with multiple drugs means studies reporting overall occurrence of rhabdomyolysis for all intoxicated patients but did not determine the incidence of rhabdomyolysis separately for each substance. Rhabdomyolysis occurrence was defined if included patients had CPK > 1000 IU/L/ or CPK > 5 × ULN^14^. Any disagreement at each stage was checked by a third researcher (AA).

### Risk of bias of included studies

The risk of bias in studies was assessed independently by two researchers (MB and MS) and disagreements were discussed and checked by the third researcher (KE). For this purpose, Newcastle–Ottawa scale (NOS) was used to assess quality of nonrandomized studies in meta-analyses and the number of stars indicated methodological quality of articles.

### Synthesis of results

The number of intoxicated patients in the studies was considered as denominator of the fraction and the number of samples containing occurrence of rhabdomyolysis was placed in the numerator. The effect size for rhabdomyolysis incidence in each subgroup was determined as pooled effect size with 95% confidence interval (CI). Weighted mean difference (WMD) was used to compare values of renal function indexes including blood urea nitrogen (BUN), creatinine (Cr), and CPK between intoxicated patients with and without rhabdomyolysis. The pooled effects size was estimated using random effect model by considering disparities between studies. Heterogeneity between studies was estimated by Cochrane Q test and I^2^ index. The type of hospitalization wards, geographical area, and multi-intoxication were considered to find the source of heterogeneity in the subgroup analysis. Publication bias was determined by Egger’s regression and Begg’s test. Sensitivity analysis was performed to assess the impact of a single study on the results. All statistical analyses for meta-analysis were done in Stata software (version 16.0; Stata Corporation).

## Results

### Study selection and characteristics

**Figure 1:**
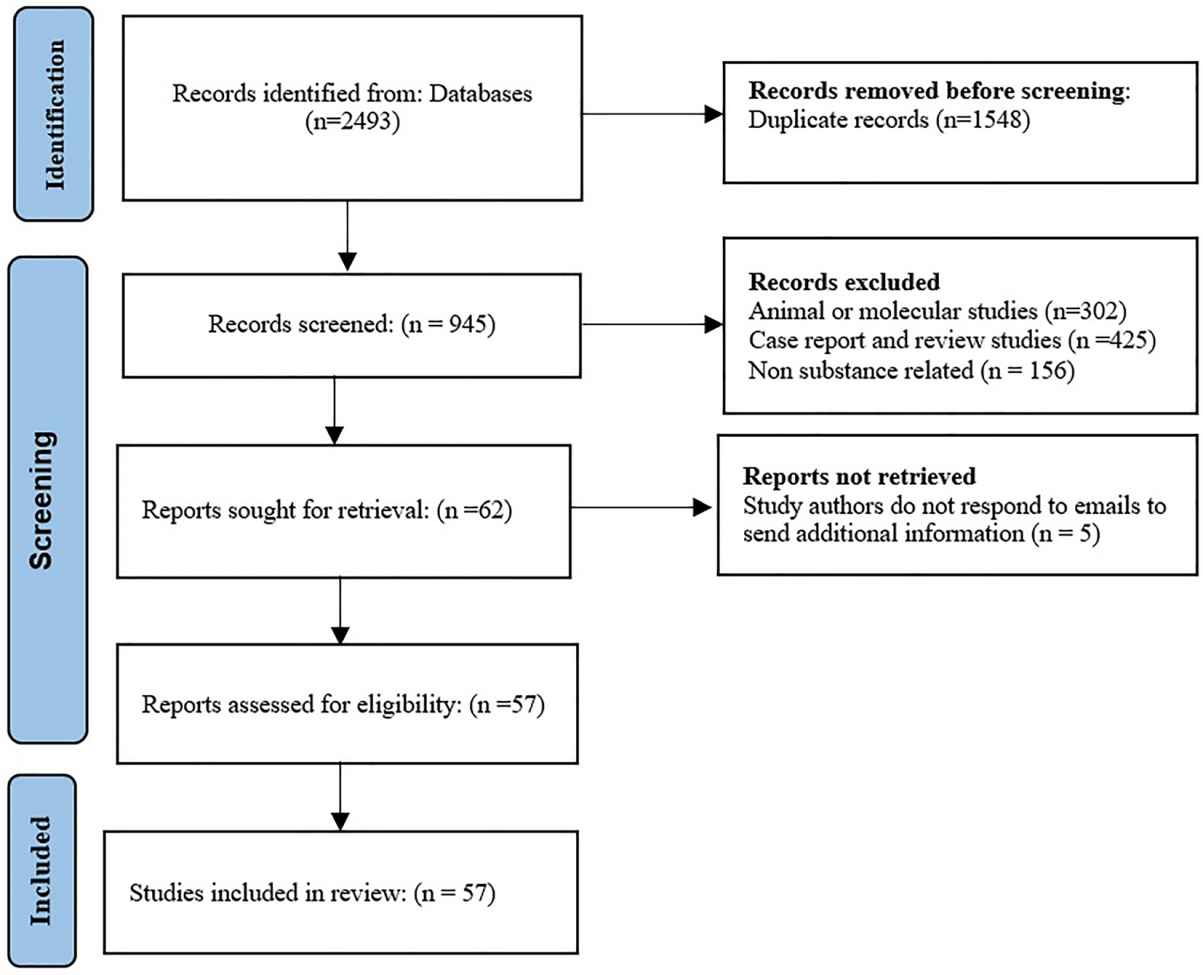
Flow diagram of the literature search for studies included in meta-analysis.

The process of study selection is depicted in the PRISMA flow diagram (Fig. 1). In the systematic search of electronic databases, 2493 nonredundant studies were found, of which 62 articles were potentially relevant. After reading their full texts, 5 articles were excluded due to lack of required data and 57 articles met the inclusion criteria for final analysis.

As described in Table 2, the percentage of hospitalized men in all included studies was high compared to women. The minimum and maximum mean age of patients was 19.4 and 46.0 years, respectively. The reported median age ranged from 19 to 50 years. A summary of included article characteristics is described in Table 2. Based on the types of psychoactive substances, a total of 57 articles containing 3,122,944 intoxicated patients were screened. Included articles were 11 studies for opioids, 6 for methadone, 11 for synthetic cannabinoids, 7 for cocaine, 17 for amphetamines, 5 for methanol, 4 for ethanol, 4 for heroin, 2 for tramadol, and 15 for multiple drugs. Most of the studies were done in the United States (n = 24), Iran (n = 13), Canada (n = 3), and Australia (n = 3).

**Table 2:** Characteristics of the studies included in the systematic review and meta-analysis.

ID	Author/reference	Year	Country continent	Design	Age*	Sex	Sample size	Poisoning drug	Participants	Multi-intoxication	Dose	Route of administration	PMH	Risk of bias
1	Aghabiklooei^15^	2014	Iran/EM	Cross-sectional	36.0 ± 15.8	(74.8%)M/(25.2%)F	322	Methadone	2007–2012/total hospitalized	No	85.91 ± 82.61 (mg)	Oral	No	*******
2	Arefi^16^	2014	Iran/EM	Cross-sectional	30.55 ± 15.8	(52%)M/(48%)F	1500	Multiple drugs/opium/alcohol/sedative	During year 2010/total hospitalized	NA	NA	Oral/IV/inhalation	No	********
3	Armenian^17^	2012	USA/America	Case series	22.42 mean (19–35)	(58.3%)M/(41.7%)F	12	AMPH/MDMA	May 30, 2010/ICU hospitalized	No	0.75 ± 0.48 (mg/l)	Oral	No	*****
4	Azarakhshi^18^	2021	Iran/EM	Retrospective	38.26 ± 25.91	(75.8%)M/(24.2%)F	227	Opium	Mar 2006–Mar 2016/total hospitalized	No	4456 ± 10,317 (mg)	Oral	NA	******
5	Brahmi^19^	2007	Tunisia/WP	Case series	21.5 mean (16–53)	(93.75%)M/(6.25%)F	16	Methanol	Dec 2003–Apr 2004/ICU hospitalized	No	250 ml (range 30–1000)	Oral	NA	******
6	Bruggisser^20^	2009	Switzerland/Europe	Retrospective	29.21 ± 12.54	(61.3%)M/(38.7%)F	220	MDMA/cocaine/AMPH	1997 and 2009/total hospitalized	No	NA	Oral/nasal/IV/inhalation	NA	*******
7	Burton^2,1^	2019	USA/America	Cross-sectional	50 median (34–60)	(47.2%)M/(52.8%)F	570,987	Opium/methadone/heroin/other opiates/narcotic	In 2010–2014/total hospitalized	Cocaine/AMPH/BDZ/aromatic analgesic	NA	NA	HTN/MDD/Psychiatric/AKI	********
8	Caldicott^2,2^	2003	Australia/WP	Case series	25 mean	(57.9%)M/(42.1%)F	19	MDMA/PMA	Jan 1999–Dec 2001/total hospitalized	BARB/THC/BDZ	NA	Oral	NA	*****
9	Chhabra^23^	2017	USA/America	Retrospective	21 median (19.5–24)	(53.6%)M/(46.4%)F	28	Ethanol/ecstasy/marijuana/LSD/cocaine	The 3-day/total hospitalized	No	NA	Oral	NA	*****
10	Dabby^24^	2006	Israel/Europe	Research report	29.5 mean (22–42)	(100%)M	6	Heroin	In 2001 and 2005/total hospitalized	No	NA	Sniffing/IV	Hepatitis C	*****
11	Forrester^25^	2021	USA/America	Retrospective	23 mean (12–67)	(74.2%)M/(25.8%)F	454	Synthetic cannabinoids	During 2010/total hospitalized	Alcohol/alprazolam/cocaine/acetaminophen	NA	Inhalation/oral	NA	******
12	Gilley^26^	2021	USA/America	Prospectively	16 to 19	(91%)M/(9%)F	75	Synthetic cannabinoids	Sep 2008–Feb 2011/total hospitalized	NA	NA	NA	NA	******
13	Greene^27^	2003	England/Europe	Case series	19.4 mean (17–23)	(85.7%)M/(14.3%)F	7	Ecstasy/MDMA	One day between the hours of 6 and 8 AM/total hospitalized	No	0.63 ± 0.83 (mg/l)	Oral	NA	*****
14	Halpern^28^	2010	Israel/Europe	Prospective	24.2 ± 6.3	(63%)M/(37%)F	52	Ecstasy/MDMA	Aug 2002–Feb 2003/total hospitalized	Alcohol/opiates/cocaine/cannabis/BDZ/LSD	NA	Oral	NA	******
15	Hermanns-Clausen^29^	2012	Germany/Europe	Retrospective	19 median (14–30)	(86.2%)M/(13.8%)F	29	Synthetic cannabinoids	Sep 2008–Feb 2011/total hospitalized	AMPH/BDZ/lorazepam/MAMP	10.99 ± 13.91 (ng/ml)	NA	NA	******
16	Heyerdahl^3,0^	2008	Norway/Europe	Prospective cross-sectional	≥ 16 years	–	947	Methanol/AMPH/opioids/ethanol/cocaine	Apr 2003–Mar 2004/total hospitalized	Codeine/paracetamol/BDZ/opioid	NA	Oral	NA	*******
17	Imam^3,1^	2013	USA/America	Case series	35.4 mean (28–42)	(100%)M	5	AMPH	May–December 2011/ICU hospitalized	Cannabinoids/cocaine	NA	Ingestion/smoke	Bipolar/ADHD/MDD/anxiety	*****
18	Isoardi^32^	2019	Australia/WP	Retrospective/observational	31 median (16–68)	(71%)M/(29%)F	329	MAMP	During 2016/total hospitalized	Alcohol/cannabis/BDZ/heroin/MDMA	NA	IV/inhalation/oral	NA	******
19	Isoardi^33^	2020	Australia/WP	Prospective/observational	32 median (28–31)	(85.4%)M/(14.6%)F	48	MAMP	Dec 2017–Sep 2018/total hospitalized	Opioid/aspirin/ethanol/LSD/GHB/marijuana	NA	IV/inhalation/oral	NA	*******
20	Kaewput^34^	2020	USA/America	Retrospective cohort	37.9 ± 18.3	(69.5%)M/(30.5%)F	603	Methanol	From 1993–2014/total hospitalized	No	NA	oral	ObesityHTN/CKD/DM	******
21	Kamijo^35^	2014	Japan/WP	Retrospective	28.4 ± 8.4	(82%)M/(18%)F	518	Synthetic cannabinoid	Jan 2006–Dec 2012/total hospitalized	Alcohol/BDZ psychotropic drug	NA	Inhalation/ingestion/sniffing/anal	NA	******
22	Kasper^36^	2018	USA/America	Case Reports	29 median (23.5–36.5)	(80%)M/(20%)F	56	Synthetic cannabinoids	Apr–May 2015/total hospitalized	Cannabinoids/BDZ cocaine/AMPH	NA	NA	HTN/seizure/mental illness	******
23	Katz^37^	2016	USA/America	Case series	22.36 mean (13–50)	(63.6%)M/(36.4%)F	11	Synthetic cannabinoids	Apr 2015/ICU hospitalized	Caffeine/morphine/midazolam/ethanol/AMPH/lorazepam	NA	NA	Hepatitis/bipolar/epilepsy	*****
24	Khoshideh^3,8^	2017	Iran/EM	Cross-sectional	37.69 ± 5.87	(82.2%)M/(17.8%)F	354	Methadone/tramadol/opium/cocaine/heroin	2014/ICU hospitalized	No	NA	NA	NA	******
25	Kitchen^3,9^	2021	Canada/America	Cross-sectional	44.7 mean (31–54)	(57.3%)M/(42.7%)F	3552	Opium/heroin/methadone/synthetic/semisynthetic	Jan 2010–Dec 2019/total hospitalized	No	NA	Oral/inhalation/IV	NA	******
26	Kourouni^4,0^	2020	USA/America	Case series	41 (25–59)	(80%)M/(20%)F	30	Synthetic cannabinoid	2014–2016/ICU hospitalized	Cannabinoids/BDZ cocaine/opioid//methadone	NA	Ingestion	Psychiatric illness/personality disorder	*****
27	Lam^4,1^	2010	China/WP	Cross sectional	38 (30–49)	(45.7%)M/(54.3%)F	265	Alcohol BDZ/TCA	Jan 2000–May 2008/ICU hospitalized	No	NA	NA	Psychiatric disease/MDD	******
28	Lavergne^12^	2016	USA/America	Retrospective	43.7 mean (43.7–44.9)	–	1745	Ethanol/methanol/cocaine/methadone	1993–2014/total hospitalized	No	NA	NA	NA	******
29	Lund^42^	2012	Norway/Europe	Cross sectional	36 (16–93)	(55.5%)M/(44.5%)F	1065	Ethanol/opioids/cocaine/AMPH/BDZ	Apr 2008–Apr 14, 2009/total hospitalized	BDZ/paracetamol/ethanol/anabolic steroids	NA	NA	NA	******
30	Mehrpour^4,3^	2020	USA/America	Retrospective toxic registry	41.9 ± 16.6	(60.2%)M/(39.8%)F	973	Methadone	Jan 2010–Dec 2017/total hospitalized	NA	111.34 ± 121.78 (mg)	Oral/parenteral	NA	*******
31	Melli^44^	2005	USA/America	Prospective	47 median (4–95)	(68.2%)M/(31.8%)F	475	Illicit drugs/alcohol	Jan 1993–Dec 2001/total hospitalized	No	NA	NA	No	******
32	Monte^45^	2017	USA/America	Prospective	25 median (18–36)	(84.1%)M/(15.9%)F	353	Synthetic cannabinoids	Jan 2010–Jul 2015/total hospitalized	Marijuana/sympathomimetic	NA	Inhalation	NA	******
33	Mozafari^46^	2016	Iran/EM	Cross sectional	32.65 ± 14.4	(59%)M/(41%)F	310	AMPH/opium/methanol/tramadol/methadone	Feb 2014–Feb 2015/ICU hospitalized	No	NA	NA	No	*******
34	Morrow^47^	2019	Canada/America	Retrospective cohort	48 median (32–61)	(53.5%)M/(46.5%)F	2554	Opium/heroin/methadone/other opioids	2006 to 2015/total hospitalized	No	NA	NA	Psychiatric /Pneumonia/HIV/cancer	*******
35	Ng^48^	2019	Hong Kong/WP	Retrospective	36.5 median (27.5–53.2)	(56.3%)M/(43.7%)F	270	Ethanol/heroin/cocaine/AMPH/cannabis/tramadol	Jan 2007–Dec 2016/ICU hospitalized	NA	NA	Oral/inhalation/parenteral/insufflation	Psychiatric/schizophrenia,	*****
36	Nicol^4,9^	2015	Canada/America	Retrospective case series	24 median (14–52)	(81.5%)M/(18.5%)F	27	PMA/MDMA	Jun 2011–Apr 2012/total hospitalized	AMPH/cocaine/MAMP	2.70 ± 1.72 (mg/l)	Oral	NA	****
37	O’Connor^5,0^	2015	USA/America	Retrospective chart review	32 median (25–42)	(74%)M/(26%)F	89	Synthetic cathinone’s/MAMP/cocaine	Jan 2010–Jan 2013/total hospitalized	No	NA	Ingestion	NA	********
38	Oladunjoye^5,1^	2020	USA/America	Cross sectional	44.6 ± 0.1	(55%)M/(45%)F	2,528,751	Opium/heroin/methadone	Jan 2010–Dec 2014/total hospitalized	No	NA	NA	Hepatitis C	*******
39	Pajoumand^5,2^	2018	Iran/EM	Retrospective cross-sectional	34.9 ± 14.5	(77%)M/(23%)F	315	Alcohol/opium	Jul 2011 and Jul 2017/total hospitalized	No	NA	NA	No	******
40	Rahimi^53^	2018	Iran/EM	Retrospective cross-sectional	32.9 ± 10.9	(77%)M/(23%)F	226	AMPH	Apr 2011–Mar 2014/total hospitalized	No	1.64 ± 1.59(gram)	Oral/inhalation/ injection	NA	*******
41	Rahimi^5,4^	2022	Iran/EM	Prospective	33 median (25, 49)	(92.7%)M/(7.3%)F	165	Opium/heroin/methadone/tramadol	Sep 2019–Mar 2020/ICU hospitalized	NA	NA	Oral/inhalation	DM/COPD/CHF/CVD/seizure	******
42	Richards^9^	2020	USA/America	Retrospective review	46 ± 15	(69%)M/(31%)F	215	Cocaine	Jul 2012–Jul 2017/total hospitalized	AMPH ethanol	NA	NA	CVD/psychiatric/neurological/GI/GU	********
43	Richards^55^	2020	USA/America	Retrospective review	Median 43 ± 13	(71%)M/(29%)F	957	MAMP AMPH	Jul 2012–Jul 2017/total hospitalized	Cocaine ethanol	NA	NA	Psychiatric/endocrine/GI/GU/ neurological	********
44	Riederer^56^	2017	USA/America	Case reports	70.6 (19–65) 27.4 (13–18)	(83.1%)M/(16.9%)F	456	Synthetic cannabinoids	Jan 2010–Nov 2015/total hospitalized	No	NA	NA	NA	*******
45	Ridpath^57^	2014	USA/America	Case report	21 median (16–29)	(41%)M/(59%)F	22	Alcohol/cocaine/ MDMA	Sep 2013 over the 3-day/total hospitalized	Synthetic club drug/marijuana/other drugs	NA	NA	NA	*****
46	Sheibani^11^	2021	Iran/EM	Observational	33 (24–46) 63 (38–71)	(75.9%)M/(24.1%)F	245	Methadone	Jun 2018–Feb 2019/ICU hospitalized	No	NA	Oral	No	******
47	Sporer^5,8^	2001	USA/America	Retrospective chart review	34	(85.2%)M/(14.8%)F	27	Heroin	Aug 1994–Dec 1998/total hospitalized	No	NA	NA	NA	*****
48	Taheri^59^	2013	Iran/EM	Cross-sectional	36.2 ± 14.50	(91.5%)M/(8.5%)F	82	Alcohol/narcotic/psychotropic	During a 6-month period in 2012/total hospitalized	No	NA	NA	NA	******
49	Talaie^8^	2007	Iran/EM	Cross sectional	32.43 ± 14.31	(64.6%)M/(35.4%)F	181	Opium/alcohol/heroin	Sep 2004–Sep 2005/ICU hospitalized	BZD/TCA/carbamazepine/phenobarbital	NA	NA	No	******
50	Talaie^6,0^	2019	Iran/EM	Cross-sectional	–	(71.8%)M/(28.2%)F	170	Opioid/methadone/stimulants	Jun 2015–Mar 2017/ICU hospitalized	Opioid/stimulants/TCA/CO	NA	NA	No	******
51	Talaie^6,1^	2020	Iran/EM	Prospective/observational/cohort	39.43 ± 16.27	(67.4%)M/(32.6%)F	184	Methadone/tramadol/AMPH/opiate	Oct 2019–Aug 2020/ICU hospitalized	NA	NA	NA	Neurological/CVA/psychiatric	*******
52	Tatusov^6,2^	2019	USA/America	Retrospective case series	47 median (32–54)	(83%)M/(17%)F	23	Synthetic cannabinoids	Jan–Dec 2015/ICU hospitalized	Alcohol/BDZ/opioid/AMPH/cocaine/phencyclidine	NA	NA	NA	*****
53	Thongprayoon^4^	2021	USA/America	Retrospective cohort	37.9 ± 18.3	(69.8%)M/(30.2%)F	603	Methanol	2003–2014/total hospitalized	NA	NA	oral	DM/HTN /anemia/CVA/CKD	*******
54	Waldman^10^	2021	10 countries/Europe	Retrospective	30 median (12–88)	(76.3%)M/(23.7%)F	1015	Cocaine/cannabis/AMPH/ heroin	Oct 2013–Sep 2014/total hospitalized	No	NA	NA	NA	*******
55	Weng^63^	2022	Taiwan/WP	Retrospective	37 median (30–43.7)	(78.4%)M/(21.6%)F	379	MAMP	May 2017–Apr 2021/total hospitalized	No	NA	NA	Psychiatric/DM/HTN/cancer	******
56	West^64^	2010	USA/America	Observational case series	27 median (16–57)	(80%)M/(20%)F	55	MAMP	Jan 2001–Jul 2007/total hospitalized	Cannabinoids/BDZ/opiate/cocaine/BARB	Median 3.5 g	Oral/ingestion	NA	*****
57	Yalçın^65^	2019	Turkey/Europe	Retrospective	26.8 ± 7.5	(92.6%)M/(7.4%)F	340	Synthetic cannabinoids	Feb–May 2016/total hospitalized	Cannabis/alcohol	NA	NA	Psychiatric illness	*******

*(Mean ± SD), Mean or Median (IQR).

*EM* Eastern Mediterranean, *WP* Western Pacific, *NA* Not Available, *IV* Intravenous, *PMH* Past Medical History, *PMA* Paramethoxyamphetamine, *BARB* Barbiturate, *AMPH* Amphetamine, *MAMP* Methamphetamine, *MDMA* 3,4-methylenedioxyamphetamine, *METH* Methylamphetamine, *THC* Tetrahydrocannabinol, *BDZ* Benzodiazepines, *LSD* Lysergic acid diethylamide, *GHB* Gamma-Hydroxybutyrate, *CO* Carbon Monoxide, *ADHD* Attention-Deficit/Hyperactivity Disorder, *CKD* Chronic Kidney Disease, *DM* Diabetes Mellitus, *COPD* Chronic Obstructive Pulmonary Disease, *CHF* Congestive Heart Failure, *CVD* Cardiovascular Disease, *HTN* Hypertension, *GI* Gastrointestinal, *GU* Genitourinary.

### Pooled estimate of rhabdomyolysis occurrence

**Figure 2:**
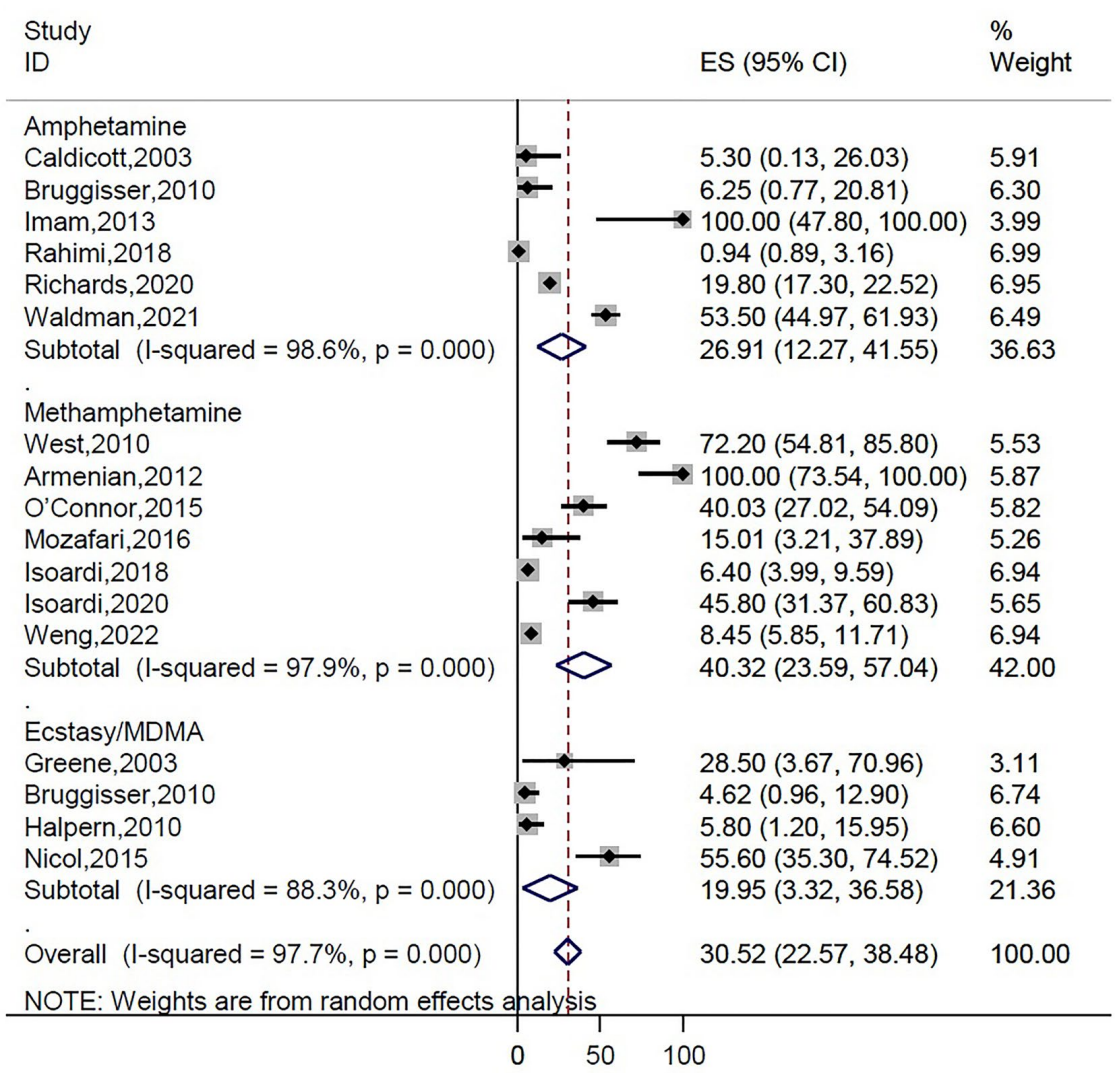
Pooled incidence of rhabdomyolysis based on the types of amphetamines intoxication.

**Figure 3:**
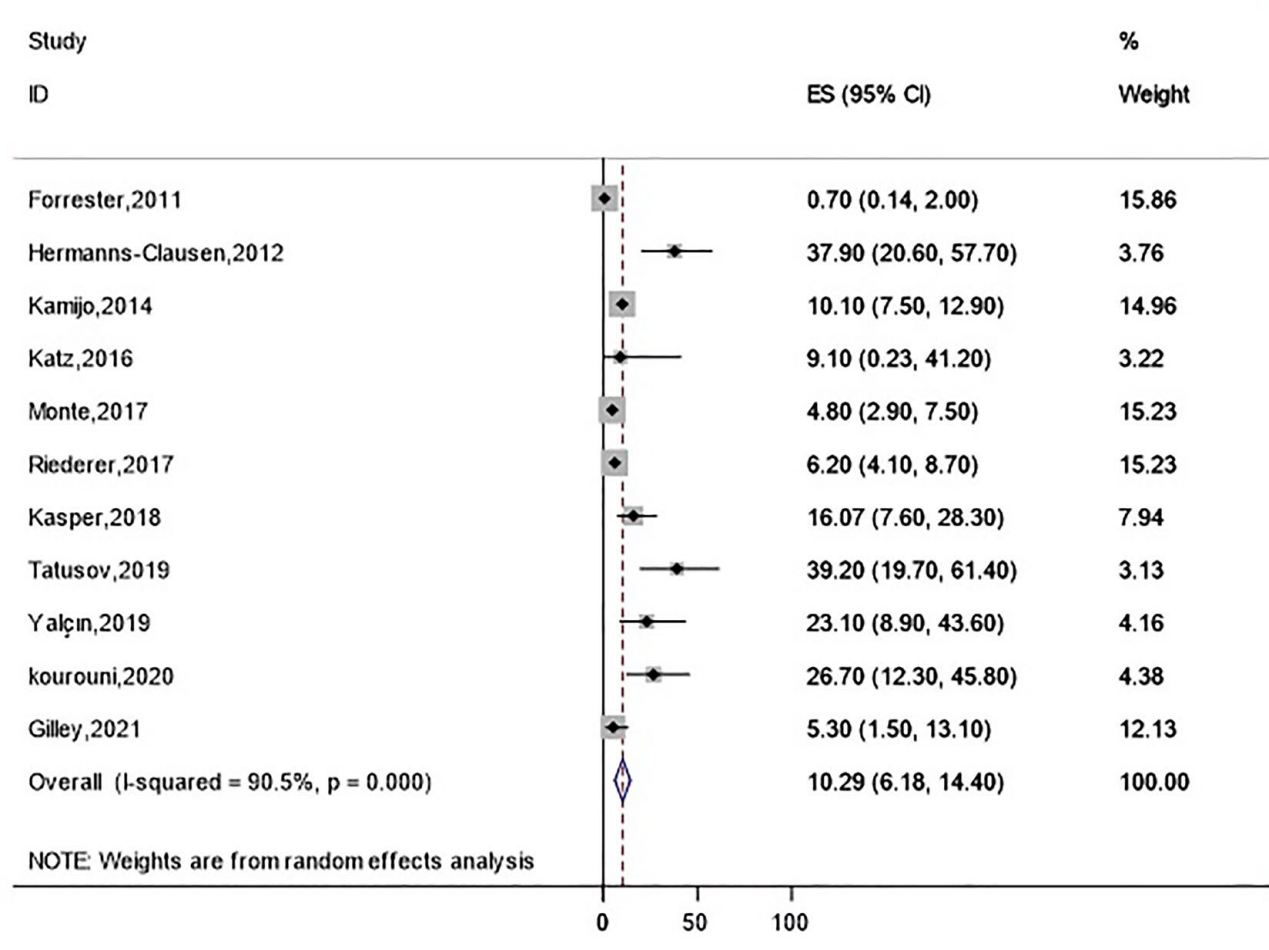
Pooled incidence of rhabdomyolysis for synthetic cannabinoid intoxication.

**Figure 4:**
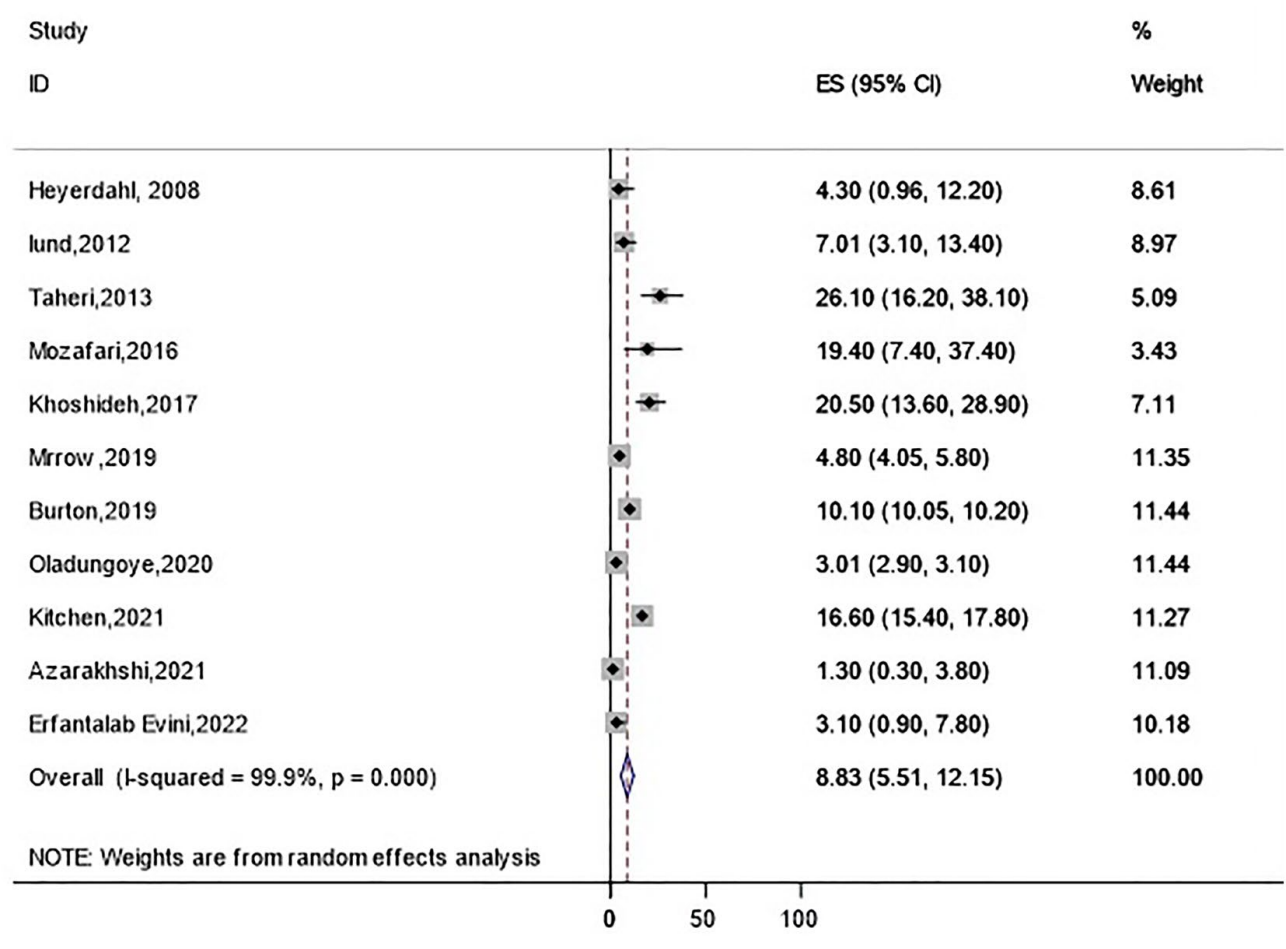
Pooled incidence of rhabdomyolysis for opioid intoxication.

The highest incidence of rhabdomyolysis occurrence was observed in heroin intoxication (57.2 [95% CI 22.6–91.8]), followed by amphetamines (30.5 [95% CI 22.6–38.5]) (Fig. 2), cocaine (26.6 [95% CI 11.1–42.1]), tramadol (17.07 [95% CI 10.6–23.5]), methadone (16.1 [95% CI 9.6–22.5]), synthetic cannabinoids (10.3 [95% CI 6.2–14.4]) (Fig. 3), and opioid (8.8 [95% CI 5.5–12.1]) (Fig. 4) (Table 3). The pooled incidence of rhabdomyolysis was low for intoxication with methanol (2.0 [95% CI 0.5–3.5]) and ethanol (3.0 [95% CI 0.3–5.7]) when compared with other psychoactive substances. In the amphetamine family, the pooled estimate of rhabdomyolysis for methamphetamine was 40.3 (95% CI 23.6–57.04), for amphetamine was 26.9 (95% CI 12.2–41.5), and for ecstasy was 19.9 (95% CI 3.3–36.5).

**Table 3:** Overall and subgroup incidence of rhabdomyolysis according to psychoactive substances intoxication.

Drugs	Subgroup	Number of studies	Pooled ES (95%CI)	Heterogeneity		Publication bias (p-value)	
				I^2^ (%)	P-value		
Opioid	Total	11	8.8 (5.5–12.1)	99.9%	< 0.001	Begg’s	Egger’s
	Subgroup analysis					0.76	0.49
	Type of admission						
	ICU	3	13.6 (2–27.8)	91.0%	0.001		
	Total wards	8	8.1 (4.3–11.8)	99.9%	< 0.001		
	Multi-intoxication						
	Yes	4	6.4 (3.1–10.6)	77.7%	< 0.001		
	No	7	10.5 (4.8–17.9)	99.3%	< 0.001		
	Geographic area						
	America	4	7.9 (3.2–14.4)	99.9%	< 0.001		
	Eastern Mediterranean	5	11.4 (2.5–25.2)	29.7%	0.223		
	European	2	5.9 (2.8–9.9)	0.0%	0.861		
Methadone	Total	6	16.1 (9.6–22.5)	93.4%	< 0.001	0.042	0.020
	Subgroup analysis						
	Type of admission						
	ICU	3	19.3 (1.6–37.0)	92.6%	< 0.001		
	Total wards	3	16.5 (4.6–28.3)	95.9%	< 0.001		
	Multi-intoxication						
	Yes	1	4.4 (3.1–5.8)	–	–		
	No	5	20.6 (10.3–30.8)	93.2%	< 0.001		
	Geographic area						
	America	2	5.5 (4.2–7.1)	84.9%	0.01		
	Eastern Mediterranean	4	16.6 (6.7–29.5)	38.5%	0.181		
Synthetic cannabinoid	Total	11	10.3 (6.2–14.4)	90.5%	< 0.001	0.073	0.015
	Subgroup analysis						
	Type of admission						
	ICU	3	25.0 (9.0–41.1)	51.6%	0.13		
	Total wards	8	8.2 (4.2–12.3)	91.8%	< 0.001		
	Multi-intoxication						
	Yes	10	13.2 (6.5–21.7)	92.7%	< 0.001		
	No	1	6.1 (3.6–9.6)	–	–		
	Geographic area						
	America	8	9.3 (3.7–16.6)	0.0%	0.568		
	European	2	30.7 (18.9–43.8)	0.0%	0.579		
	Western pacific	1	10.1 (7.6–12.9)	–	–		
Amphetamines	Total	17	30.5 (22.6–38.5)	97.7%	< 0.001	Begg’s	Egger’s
	Subgroup analysis					0.099	0.104
	Type of admission						
	ICU	3	71.5 (12.8–100)	96.8%	< 0.001		
	Total wards	14	22.7 (15.5–9.9)	97.1%	< 0.001		
	Multi-intoxication						
	Yes	8	31.6 (16.8–48.4)	95.5%	< 0.001		
	No	9	24.1 (8.3–44.2)	97.1%	< 0.001		
	Geographic area						
	America	6	65.6 (35.6–90.5)	79.7%	< 0.001		
	Eastern Mediterranean	2	15.0 (3.2–37.8)	0.0%	0.541		
	European	5	16.3 (0.4–44.6)	78.3%	0.001		
	Western pacific	4	13.6 (4.5–26.3)	55.7%	0.079		
Methanol	Total	5	2.0 (0.5–3.5)	74.0%	0.004	0.086	0.003
	Subgroup analysis						
	Type of admission						
	ICU	2	21.0 (6.1–35.9)	0.0%	0.75		
	Total wards	3	1.75 (0.5–2.4)	76.7%	0.014		
	Multi-intoxication						
	Yes	1	2.3 (1.3–3.8)	–	–		
	No	4	3.4 (0.2–8.9)	89.1%	0.001		
	Geographic area						
	America	3	1.6 (0.6–3.1)	0.0%	0.927		
	Eastern Mediterranean	2	21.2 (8.2–37.4)	0.0%	0.889		
Ethanol	Total	4	3.0 (0.3–5.7)	65.8%	0.03	0.497	0.305
	Subgroup analysis						
	Multi-intoxication						
	Yes	2	1.6 (0.5–3.2)	0.0%	0.42		
	No	2	2.5 (0.6–5.3)	55.7%	0.13		
	Geographic area						
	America	1	5.1 (2.9–8.1)	–	–		
	Eastern Mediterranean	1	33.3 (4.3–77.7)	–	–		
	European	2	1.6 (0.5–3.2)	89.2%	< 0.001		
Cocaine	Total	7	26.6 (11.1–42.1)	95.3%	< 0.001	Begg’s	Egger’s
	Subgroup analysis					0.881	0.614
	Type of admission						
	ICU	1	40.0 (0.5–79.5)	–	–		
	Total wards	6	25.4 (9.1–41.7)	96.1%	< 0.001		
	Multi-intoxication						
	Yes	2	23.2 (17.6–29.1)	0.0%	0.535		
	No	5	28.6 (9.3–52.5)	69.3%	0.01		
	Geographic area						
	America	3	29.9 (21.0–39.6)	0.0%	0.573		
	European	2	17.3 (0.05–56.6)	85.5%	0.001		
	Eastern Mediterranean	1	40.0 (5.27–85.3)	–	–		
Heroin	Total	4	57.2 (22.6–91.8)	94.6%	< 0.001	0.174	0.785
	Subgroup analysis						
	Type of admission						
	ICU	1	100.0 (39.7–100)	–	–		
	Total wards	3	40.6 (7.1–79.5)	90.4%	< 0.001		
	Geographic area						
	America	1	7.4 (0.9–24.3)	–	–		
	Eastern Mediterranean	1	100 (39.7–100)	–	–		
	European	2	50.3 (41.5–59.2)	0.0%	0.391		
Tramadol	Total	2	17.07 (10.6–23.5)	0.0%	0.61	0.317	–
Multiple drug	Total	15	29.7 (19.8–39.5)	98.6%	< 0.001	0.458	0.712
	Subgroup analysis						
	Type of admission						
	ICU	6	30.5 (14.4–49.4)	98.1%	< 0.001		
	Total wards	9	26.7 (14.6–40.9)	98.4%	< 0.001		
	Multi-intoxication						
	Yes	8	25.3 (11.7–42.0)	98.3%	< 0.001		
	No	7	31.6 (20.2–44.1)	97.1%	< 0.001		
	Geographic area						
	America	4	30.4 (19.6–42.3)	0.0%	0.558		
	Eastern Mediterranean	7	34.4 (19.5–51.0)	92.5%	< 0.001		
	European	2	34.4 (31.8–37.1)	97.5%	< 0.001		
	Western pacific	2	16.5 (13.5–19.8)	54.2%	0.139		

### Subgroup analysis

#### Hospitalization ward and multi-intoxication

The pooled effect size in the subgroup of ICU patients was higher than in the total wards (Table 3). Incidence of rhabdomyolysis in ICU patients and total wards was respectively 13.6% vs 8.1% for opioids, 19.3% vs 16.5% for methadone, 25% vs 8.2% for synthetic cannabinoids, 40.0% vs 25.4% for cocaine, 71.5% vs 22.7% for amphetamines, 21% vs 1.75% for methanol, 100% vs 40.6% for heroin, and 30.5% vs 26.7% for multiple drug poisoning. In addition, pooled effect size in the subgroup of total wards was influenced by the severity of intoxication, which was different in the included studies. In comparison with multi-intoxication subgroup, pooled effect size of rhabdomyolysis occurrence was high in mono-intoxication subgroup except for synthetic cannabinoids (6.1% vs 13.2%) and amphetamines (24.1% vs 31.6%).

#### Geographic area

As it was showed in Table 3, according to the subgroup analysis based on the geographic area, the highest incidence of rhabdomyolysis occurrence was related to amphetamines in American region (65.6% [35.6–90.5]) and synthetic cannabinoids in European region (30.7% [18.9–43.8]), whereas the highest incidence of rhabdomyolysis occurrence in Eastern Mediterranean was related to other psychoactive substances. Subgrouping by geographic region reduced heterogeneity between studies.

#### Pooled mean effect size of renal function indexes

Table 4 shows the comparison of mean effect size of renal function indexes in patients with and without rhabdomyolysis. Accordingly, the value of BUN (WMD = 8.78, p = 0.002), Cr (WMD = 0.44, p < 0.001), and CPK (WMD = 2590.9, p < 0.001) was significantly high in patients with rhabdomyolysis compared to those patients without rhabdomyolysis.

**Table 4:** Weighted mean difference of renal indices according to rhabdomyolysis.

Renal function index	Number of studies	Weighted Mean Difference (%95)	p-value group	Heterogeneity test		Publication bias	
				I^2^ (%)	p-value	Egger’s	Begg’s
BUN (mg/dl)	3	8.78 (7.87–9.69)	0.002	25.13	< 0.394	0.117	0.274
Cr (mg/dl)	4	0.44 (0.22–0.65)	< 0.001	97.81	< 0.001	0.738	0.993
CPK (u/l)	4	2590.9 (1973.8–3208.1)	< 0.001	98.72	< 0.001	0.348	0.497

*BUN* Blood Urea Nitrogen, *Cr* Creatinine, *CPK* Creatine Phosphokinase.

#### Publication bias and sensitivity analysis

Table 3 represent specified p-values related to the publication bias in each type of psychoactive substances with Begg’s and Egger’s test, indicating that there was no publication bias in most of the intoxications. Sensitivity analysis was performed for all tests applied for meta-analysis and the results showed that none of the pooled effect size was influenced by a single study.

## Discussion

The current study was a systematic review and meta-analysis of clinical data related to the incidence of rhabdomyolysis among intoxicated patients with psychoactive substances. To the best of our knowledge, this was the first systematic review conducted on rhabdomyolysis occurrence in psychoactive substance intoxication. Our results showed that pooled effect size for all categories of psychoactive substances was high in the subgroup of ICU patients compared with total wards. Also, intoxication with heroin (~ 100) and amphetamine (~ 71.5) showed the highest effect size for occurrence of rhabdomyolysis in ICU patients. In a study in Iran on 227 poisoned patients with refined opium extract, the majority of them (75.8%) were male. However, it has been documented that females have higher mitochondrial mass in skeletal muscle with greater oxidative phosphorylative capacities and therefore have greater protection against rhabdomyolysis^66,67^.

A broad range of neurological complications affecting both central nervous system (CNS) and peripheral nervous system (PNS) are encountered in heroin abusers^24^. CNS lesions include brain hypoxia and seizure, spongiform leukoencephalopathy, stroke, and myelopathy^68^ while PNS involvement commonly manifests as compressive neuropathy or focal rhabdomyolysis^69^. The etiology of this acute PNS complication is unclear. Some studies found immunological causes in patients who developed rhabdomyolysis. Also, mechanical trauma is considered a potential mechanism of focal nerve injury and localized rhabdomyolysis in heroin abusers^70^. The effect size of rhabdomyolysis in ICU patients with heroin intoxication (100 [95% CI 39.7–100]) was the highest in our study.

The second highest effect size of rhabdomyolysis in ICU patients was observed in intoxication with amphetamines (71.5 [95% CI 12.8–100]). The etiology of amphetamine-induced rhabdomyolysis has traditionally been attributed to agitation and/or physical restrain with intense isometric muscle contraction. However, many patients who use amphetamines are not agitated or restrained but experience rhabdomyolysis. Some of the indirect mechanisms and cofactors are gender difference (males at higher risk), monoamine receptor polymorphisms, cocaine and sedative co-injection, seizure, sepsis, and hyperthermia^55^. Hyperthermia is a major toxic reaction to amphetamines that can lead to rhabdomyolysis, hypotension, disseminated intravascular coagulation (DIC), and AKI. Hyperthermia occurs as a result of complex interactions between serotonin, dopamine, norepinephrine, and environmental conditions^71^. In some studies that reported a high frequency of rhabdomyolysis, there are several potential explanations for poor clinical outcomes and rhabdomyolyses like hyperthermia, concertation of amphetamines, and prolonged hypoxia. Most of the severe morbidity and mortality in these cases can be attributed to hyperthermia effects^71^.

In this study we found a higher effect size for patients with mono-intoxication than those with multi-intoxication following use of psychoactive substances. In many studies, alcohol abuse has been identified as a main cause of rhabdomyolysis^8,38^ and other studies regarding the etiology of rhabdomyolysis have reported opioid overdose as a significant contributing factor to rhabdomyolysis^8^. In this regard, Talaie et al. reported that opium poisoning is the most common cause of rhabdomyolysis (23.3%), followed by poisoning with benzodiazepines, phenobarbital, propranolol, aluminum phosphide, alcohol, and co-poisoning^8^. Also, Babak et al.in their study reported that rhabdomyolysis is mostly associated with methadone abuse, followed by opium abuse, and is more commonly correlated with poisoning in younger patients^8,38^.

## Limitations

The final analysis in our study was substantially limited in the number and quality of studies available. Our review only included the published studies but we tried our best to contact researchers and obtain more information about their studies. Also, few studies in our review reported seizure induced by psychoactive intoxication that may contribute to rhabdomyolysis in intoxicated patients. The next limitation is that we had disparities in the distribution of intoxication severity in total wards subgroup affecting the effect size of the study. Furthermore, distribution of type of psychoactive substance in the subgroup of multiple poisoning was different between studies, which affects the effect size of each study. These limitations and lack of clarity in the studies caused high heterogeneity in our analysis. Another limitation of this study is that we searched only studies with English full text or at least English abstracts, and also subgroup analysis was not possible based on the dose and route of the substance used, blood levels of the drug, and other variables due to insufficient reported data in the studies. In addition, we did not perform any blinding process for all stages of study selection, quality assessment, and data extraction. Therefore, we propose running more comprehensive and original research in this regard to help make a better conclusion regarding the incidence of rhabdomyolysis in patients with psychoactive substance intoxication.

## Conclusion

In conclusion, this systematic review and meta-analysis revealed high incidence of rhabdomyolysis occurrence in patients with heroin and amphetamine intoxication compared to other psychoactive substances. Clinicians should anticipate this complication, monitor for rhabdomyolysis particularly in the ICU, and institute appropriate treatment protocols early in the patient’s clinical course.

## Data Availability

All data generated or analyzed during this study are available from the corresponding author on reasonable request.
